# Aberrant oscillatory dynamics during somatosensory processing in HIV-infected adults

**DOI:** 10.1016/j.nicl.2018.07.009

**Published:** 2018-07-10

**Authors:** Rachel K. Spooner, Alex I. Wiesman, Mackenzie S. Mills, Jennifer O'Neill, Kevin R. Robertson, Howard S. Fox, Susan Swindells, Tony W. Wilson

**Affiliations:** a Department of Neurological Sciences, University of Nebraska Medical Center (UNMC), Omaha, NE, USA; b Center for Magnetoencephalography, UNMC, Omaha, NE, USA; c Department of Internal Medicine, Division of Infectious Diseases, UNMC, Omaha, NE, USA; d Department of Neurology, University of North Carolina School of Medicine, Chapel Hill, NC, USA; e Department of Pharmacology and Experimental Neuroscience, UNMC, Omaha, NE, USA

**Keywords:** Sensory gating, Oscillatory power, Peak gamma frequency, Magnetoencephalography

## Abstract

While the arrival of combination antiretroviral therapy significantly decreased the prevalence of HIV-associated dementia, between 35 and 70% of all infected adults continue to develop some form of cognitive impairment. These deficits appears to affect multiple neural subsystems, but the mechanisms and extent of damage are not fully understood. In the current study, we utilized magnetoencephalography (MEG), advanced oscillatory analysis methods, and a paired-pulse somatosensory stimulation paradigm to interrogate pre-attentive inhibitory processing in 43 HIV-infected adults and 28 demographically-matched uninfected controls. MEG responses were imaged using a beamformer, and time series data were extracted from the peak voxel in grand-averaged functional brain images to quantify the dynamics of sensory gating, oscillatory power, spontaneous power, and other neural indices. We found a significantly weakened response to the second stimulation compared to the first across groups, indicating significant sensory gating irrespective of HIV-infection. Interestingly, HIV-infected participants exhibited reduced neural responses in the 20–75 Hz gamma range to each somatosensory stimulation compared to uninfected controls, and exhibited significant alterations in peak gamma frequency in response to the second stimulation. Finally, HIV-infected participants also had significantly stronger spontaneous activity in the gamma range (i.e., 20–75 Hz) during the baseline period before stimulation onset. In conclusion, while HIV-infected participants had the capacity to efficiently gate somatosensory input, their overall oscillatory responses were weaker, spontaneous baseline activity was stronger, and their response to the second stimulation had an altered peak gamma frequency. We propose that this pattern of deficits suggests dysfunction in the somatosensory cortices, which is potentially secondary to accelerated aging.

## Highlights

• Around half of all HIV-infected patients will develop cognitive impairments during their life.
• The underlying mechanisms are unknown, but inhibitory deficits are thought to be critical.
• We examined oscillatory activity using MEG in 71 HIV-infected and uninfected adults.
• Gamma oscillatory dynamics were significantly disturbed in HIV-infected adults.

## 1 Introduction

Since the advent of combination antiretroviral therapy (cART), the prevalence of HIV-associated dementia (HAD) has significantly decreased, while that of other forms of HIV-associated neurocognitive disorders (HAND) has remained largely the same or even increased (Antinori et al., 2007; Cysique and Brew, 2009; Heaton et al., 2010; Heaton et al., 2011; Robertson et al., 2007). Specifically, 35–70% of all HIV-infected individuals continue to develop at least some form of cognitive impairment, and this has a major economic and societal impact on the United States and worldwide (Heaton et al., 2010; Heaton et al., 2011; Robertson et al., 2007; Simioni et al., 2010).

Previous neuroimaging studies investigating the impact of HIV infection on cognitive and sensory processing suggest hyperactivation in frontal and parietal regions during cognitive tasks, and hypoactivation in primary visual cortices during visual tasks and at rest (Ances et al., 2011; Ances et al., 2009; Ances et al., 2010; Chang et al., 2004; Chang et al., 2008; Ernst et al., 2002; Wiesman et al., 2018; Wilson et al., 2013a; Wilson et al., 2017). For example, a study of resting cerebral blood flow (rCBF) revealed diminished rCBF within the lenticular nuclei and visual cortex for HIV-infected participants compared to uninfected controls. Further, this reduction in rCBF was shown in both impaired and unimpaired participants with HIV compared to controls, suggesting that at least some neurobiological changes in blood flow precede neuropsychological deficit (Ances et al., 2009). A similar study using fMRI but focusing on the interaction between age and HIV-infection revealed lower basal CBF, greater task-related changes in CBF, and task-related decreases in the blood‑oxygenation-level-dependent (BOLD) fMRI signal in HIV-infected participants compared to uninfected controls. No interactions with age occurred in this sample, but the task-related responses in the HIV-infected group were equivalent to controls who were 15–20 years older, supporting an accelerated aging perspective in neuroHIV (Ances et al., 2010). Several studies have also used fMRI to examine activation during demanding cognitive tasks. For example, Chang and colleagues found that HIV-infected adults showed similar task performance relative to uninfected controls during a set of progressively more difficult visual attention tasks, but that load-dependent activation increases were greater in the prefrontal and parietal regions of HIV-infected participants relative to controls (Chang et al., 2004; Chang et al., 2008). In sum, fMRI and rCBF studies of HIV-infection have shown hyper-activation in parietal and prefrontal cortices during cognitively-demanding tasks, and hypo-activation in visual regions during visual tasks and at rest. However, to date, most studies have either focused on the resting-state or conducted visually-based attention tasks, and very few studies have evaluated somatosensory processing in the HIV-infected brain.

Sensory gating is a neurophysiological phenomenon whereby the response to the second stimulus in a pair of identical stimuli is attenuated. This attenuation is thought to reflect the capacity of the CNS to filter less relevant information and preserve resources for behaviorally-relevant stimuli (Cromwell et al., 2008). Substantial evidence suggests that patients with cerebral palsy, schizophrenia, bipolar disorder and even healthy older adults exhibit impaired gating of auditory and/or somatosensory stimuli, which is thought to reflect an impairment in bottom-up, pre-attentive inhibitory processing (Cheng et al., 2016a; Kurz et al., 2018; Light and Braff, 1999; Spooner et al., 2018; Thoma et al., 2017). Interestingly, no study to date has evaluated gating in the somatosensory system of HIV-infected patients, which is surprising given that attentive or top-down inhibitory processing is thought to be a critical deficit (Hoare et al., 2016; Martin et al., 1992a; Martin et al., 1992b; Walker and Brown, 2017) and there is existent data suggesting that attentive and pre-attentive inhibitory processes are closely linked in healthy adults. Essentially, Cheng et al. (2016b) examined the relationship between bottom-up and top-down inhibitory processes using go/no-go tasks and paired-pulse electrical stimulation. Their findings revealed a significant negative association among somatosensory gating (a pre-attentive measure of inhibition) and behavioral performance on go/no-go tasks (an attentive measure of inhibition), such that better suppression of redundant information (i.e., smaller gating ratios) was significantly related to increased accuracy on somatosensory and auditory go/no-go tasks in healthy adults. Given these data, it is possible that previous findings of top-down attention deficits in neuroHIV may be at least partially attributable to deficits in pre-attentive inhibitory processing, but this remains speculative, as the data in this area is very limited. Thus, a key goal of the current study was to determine whether HIV-infected adults exhibit sensory gating deficits, indicative of aberrations in pre-attentive inhibitory processing.

Herein, we used a paired-pulse electrical stimulation paradigm to examine the impact of chronic HIV-infection on pre-attentive inhibitory processing in the post central gyrus. Recent studies of somatosensory processing have found that such electrical stimulation is associated with strong oscillations up to at least 75 Hz (Cheng et al., 2016b; Kurz et al., 2018; Spooner et al., 2018; Wiesman et al., 2017), and numerous cellular electrophysiology studies have shown that such high-frequency gamma oscillations are at least partially dependent on GABAergic interneuronal networks (Bartos et al., 2007; Buzsáki and Wang, 2012; Fries, 2009, Fries, 2015; Fries et al., 2007; Salkoff et al., 2015; Singer, 1999; Uhlhaas et al., 2009; Uhlhaas and Singer, 2012; Vinck et al., 2013). Thus, by quantifying gamma-frequency neural activity, we can probe local GABAergic processing and potentially identify GABA dysfunction in the neocortex. To this end, we collected high-density magnetoencephalography (MEG) from a large sample of HIV-infected and uninfected participants and applied advanced oscillatory analysis methods for hypothesis testing. Our primary hypotheses were that HIV-infected participants would have: (1) weakened cortical gamma responses to stimulation of the median nerve, (2) impaired sensory gating, (3) elevated spontaneous gamma activity prior to stimulus onset, and (4) altered peak gamma frequency in the somatosensory cortices.

## 2 Methods

### 2.1 Participants

Middle-age participants (range: 32–55 years old) were selected from a large ongoing study of the effects of aging with HIV-infection. Exclusion criteria included any medical illness affecting CNS function (other than HIV-infection/HAND), any neurological disorder (other than HAND), history of head trauma, and current substance abuse. Inclusion criteria included receiving effective cART (HIV-infected only), existence of complete MEG somatogating data (see below) and structural MRI, and complete neuropsychological assessment data. Uninfected controls were selected to match HIV-infected participants based on their ethnicity, age, sex, handedness, and educational level. In total, 43 HIV-infected adults (28 males) and 28 uninfected controls (15 males) were selected for the study. All HIV-infected participants were receiving effective combination antiretroviral therapy and had undetectable viremia. After a full description of the study was given to participants, written informed consent was obtained following the guidelines of the University of Nebraska Medical Center's Institutional Review Board, which approved the study protocol.

### 2.2 Experimental paradigm

Participants were seated in a nonmagnetic chair with their head positioned within the MEG helmet-shaped sensor array. Electrical stimulation was applied to the right median nerve using external cutaneous stimulators connected to a Digitimer DS7A constant-current stimulator system (Digitimer Limited, Letchworth Garden City, UK). For each participant, we collected at least 80 paired-pulse trials with an inter-stimulus interval of 500 ms and an inter-pair interval that randomly varied between 4500 and 4800 ms. Participants were instructed to rest with their eyes closed throughout the duration of the experimental paradigm. Each pulse generated a 0.2 ms constant-current square wave that was set to a limit of 10% above the motor threshold that was required to elicit a subtle twitch of the thumb. Note that the amplitude of stimulation did not differ between groups (*p* = .701).

### 2.3 MEG data acquisition and Coregistration with structural MRI

All recordings were performed in a one-layer magnetically-shielded room with active shielding engaged for environmental noise compensation. With an acquisition bandwidth of 0.1–330 Hz, neuromagnetic responses were sampled continuously at 1 kHz using an Elekta MEG system (Elekta, Helsinki, Finland) with 306 magnetic sensors, including 204 planar gradiometers and 102 magnetometers. Throughout data acquisition, participants were monitored using a real-time audio-video feed from inside the magnetically-shielded room. MEG data from each participant were individually corrected for head motion and subjected to noise reduction using the signal space separation method with a temporal extension (Taulu and Simola, 2006; Taulu et al., 2005) Each participant's MEG data were coregistered with structural T1-weighted MRI data prior to source space analyses using BESA MRI (Version 2.0). Structural MRI data were aligned parallel to the anterior and posterior commissures and transformed into standardized space. After beamformer analysis, each subject's functional images were also transformed into standardized space using the transform applied to the structural MRI volume and spatially resampled.

### 2.4 MEG preprocessing, time-frequency transformation, and sensor-level statistics

Cardiac artifacts were removed from the data using signal-space projection (SSP) and the projection operator was accounted for during source reconstruction (Uusitalo and Ilmoniemi, 1997). Epochs were of 3700 ms duration, with 0 ms defined as the onset of the first stimulation and the baseline being the −700 to −300 ms window. Of note, we shifted our baseline away from the period immediately preceding stimulus onset to avoid potential contamination by any anticipatory responses, although there was no evidence of such anticipatory responses in our final analyses. Epochs containing artifacts were rejected based on a fixed threshold method, supplemented with visual inspection. On average, 70 trials per participant were used for further analysis and the average number of trials accepted did not statistically differ by group.

Artifact-free epochs were transformed into the time-frequency domain using complex demodulation, and the resulting spectral power estimations per sensor were averaged over trials to generate time-frequency plots of mean spectral density. The sensor-level data per time-frequency bin was normalized using the mean power per frequency during the −700 to −300 ms baseline time period. The specific time-frequency windows used for imaging were determined by statistical analysis of the sensor-level spectrograms across all participants, which were aimed at identifying the specific post-stimulus time-frequency bins that significantly differed in power relative to the baseline. Each data point in the spectrogram was initially evaluated using a mass univariate approach based on the GLM. To reduce the risk of false positive results while maintaining reasonable sensitivity, a two stage procedure was followed to control for Type 1 error (Maris and Oostenveld, 2007). Briefly, one-sample *t*-tests were conducted on each data point and the resulting spectrogram of *t*-values was thresholded at *p* < .05 to define time-frequency bins containing potentially significant oscillatory deviations across all participants. In stage two, time-frequency bins that survived this threshold were clustered with temporally and/or spectrally neighboring bins that were also significant, and a cluster value was derived by summing all of the *t*-values of all data points in the cluster. Nonparametric permutation testing was then used to derive a distribution of cluster-values and the significance level of the observed clusters (from stage one) were tested directly using this distribution (Ernst, 2004; Maris and Oostenveld, 2007). For each comparison, at least 10,000 permutations were computed to build a distribution of cluster values. Time-frequency bins with significant responses following permutation testing were selected for the beamforming analysis (see below). Further details of this method and our processing pipeline can be found in recent papers (Spooner et al., 2018; Wiesman et al., 2017).

### 2.5 MEG Beamformer imaging and statistics

Cortical networks were imaged through an extension of the linearly constrained minimum variance vector beamformer, (Gross et al., 2001; Van Veen et al., 1997) which employs spatial filters in the time-frequency domain to calculate source power for the entire brain volume. The single images are derived from the cross spectral densities of all combinations of MEG gradiometers averaged over the time-frequency range of interest, and the solution of the forward problem for each location on a grid specified by input voxel space. Following convention, we computed noise-normalized, source power per voxel in each participant using target (i.e., task) and control (i.e., baseline) periods of equal duration and bandwidth (Hillebrand et al., 2005). MEG pre-processing and imaging used the Brain Electrical Source Analysis (Version 6.1; BESA) software. Normalized source power was computed for the selected time-frequency periods (see *Results*) over the entire brain volume per participant at 4.0 × 4.0 × 4.0 mm resolution. The resulting beamformer images were averaged across all participants to assess the neuroanatomical basis of the significant oscillatory responses identified through the sensor-level analysis, and to allow identification of the peak voxels per oscillatory response.

Voxel time series data (“virtual sensors”) were extracted from each participant's MEG data using the peak voxel coordinate identified using the grand-averaged functional image described above. To compute these virtual sensors, we applied the sensor weighting matrix derived through the forward computation to the preprocessed signal vector, which yielded a time series for the specific coordinate in source space. Note that virtual sensor extraction was done per participant, once the coordinates of interest were known. Once the virtual sensor time series were extracted, we computed the envelope of the spectral power in the frequency bin that was used in the beamforming analysis. From this time series, we computed the relative (i.e., baseline-corrected) and absolute (i.e., not baseline-corrected) response time series of each participant.

To examine the disease-related alterations in sensory processing, we conducted mixed model ANOVAs and two-tailed, independent-samples *t*-tests among somatosensory indices and groups (see *Results*). These indices included a gating ratio to quantify the degree of sensory gating (i.e., response power to stimulus 2 divided by the response power to stimulus 1; higher gating ratios reflect poorer gating), peak relative response power to the first and second stimulation, absolute baseline power from −700 to −300 ms, and peak frequency in response to the first and second stimulation.

## 3 Results

All 71 participants were able to successfully complete the MEG and MRI aspects of study. However, three participants (1 male) with HIV were excluded from the final analyses due to excessive artifacts in their MEG data and/or technical problems. The remaining 68 participants (40 participants with HIV and 28 uninfected controls) had a mean age of 44.60 years-old for HIV-infected participants and 42.35 years-old for uninfected controls This difference was not significant (*p* = .232). Thirty-seven of 40 HIV-infected participants were right-handed, while 25 of 28 controls were right-handed. Time since diagnosis, CD4, and CD4 nadir counts were collected at the time of the MEG session for participants with HIV (time since diagnosis: M = 11.8 years; CD4 = 871.95 cells/μL; CD4 nadir = 241.95 cells/μL).

### 3.1 Sensor-level analysis

**Fig. 1:**
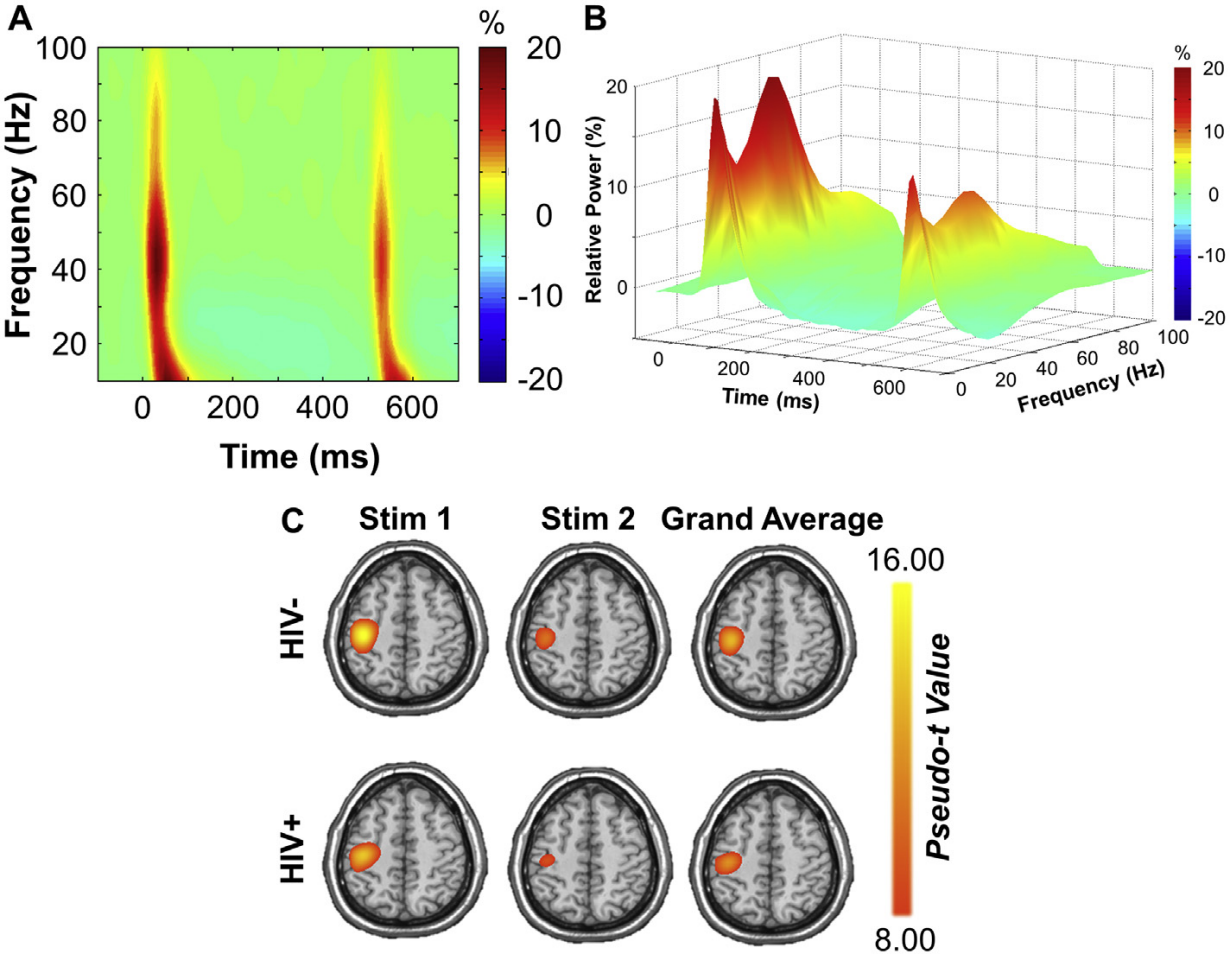
Neural responses to somatosensory stimulation of the right median nerve. (A and B): Two depictions of somatosensory spectral responses to paired-pulse electrical stimulation. (A): Time-frequency spectrogram from a MEG sensor near the sensorimotor cortices, with the x-axis denoting time (ms) and stimulation onset occurring at 0 and 500 ms. The y-axis denotes frequency (Hz). (B): A 3D spectrogram illustrating the same data as in (A) with time (ms) on the x-axis, relative power (%) on the y-axis, and frequency (Hz) on the z-axis. All signal power data is expressed as percent change from baseline (−700 to −300 ms), and the corresponding color scale bars are displayed to the right of each graphic. (C): Group averaged beamformer images (pseudo-t) for stimulation 1 (left), stimulation 2 (middle), and the group's grand-average (right). Strong increases in power were found in virtually identical areas of the contralateral hand region of the somatosensory cortex in controls (upper panel) and HIV-infected participants (lower panel). These maps were grand-averaged across both groups and stimulations to identify the peak voxel, which was followed by virtual sensor extraction and additional analyses.

Robust broadband (10–90 Hz) synchronizations were found in many sensors near the sensorimotor and parietal regions during the first 100 ms after the onset of each electrical stimulation (*p* < .001, corrected; Fig. 1). It was apparent that higher frequency, gamma range responses were much stronger during the first 50 ms after stimulus onset. Thus, we focused our beamformer analyses on the higher 20–75 Hz frequency range and utilized two 50 ms time intervals in which the neural response to stimulation was the strongest (0–50 ms and 500–550 ms). Note that our main analyses were limited on the low end to 20 Hz, as this was the lowest frequency that we could precisely resolve at 50 ms. Additionally, we restricted our analyses to 75 Hz on the high end because relative power sharply decreased thereafter, especially in response to the second stimulus.

### 3.2 Voxel-based and virtual sensor analyses

Beamformer images revealed peak responses in the contralateral somatosensory hand region of the post central gyrus, with virtually identical peak locations in response to the first and second stimulations for controls and HIV-infected participants (Fig. 1C). As described in the methods, these images were grand-averaged across all participants and both stimulations, and virtual sensor data were extracted from the peak voxel. We next computed the baseline-corrected (i.e., relative) power envelope for the 20–75 Hz band, and these data underwent statistical analysis.

### 3.3 Disease-related alterations in somatosensory processing, gating, and spontaneous activity

**Fig. 2:**
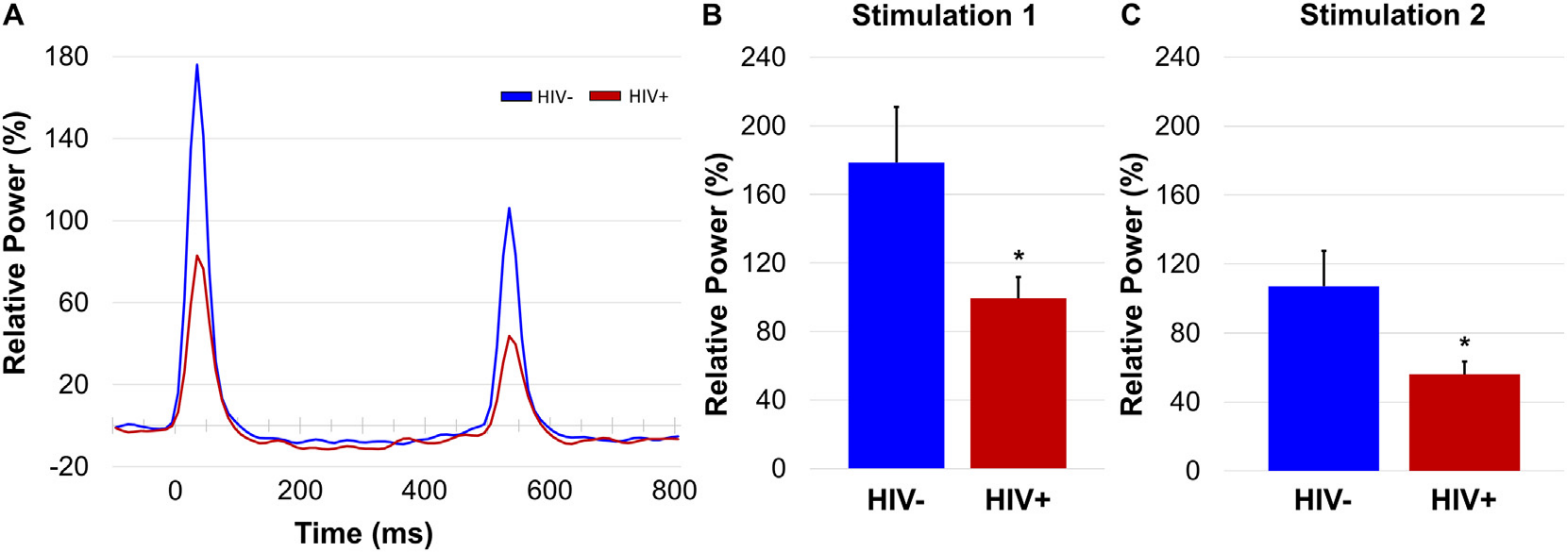
Reduced response power to somatosensory stimulation in HIV-infected participants. (A): Disease-related power differences in response to each electrical stimulation were clearly seen in the relative power envelope for the 20–75 Hz band. (B): Independent samples *t*-tests revealed significantly reduced response power to stimulation 1 and (C) 2 in HIV-infected participants (shown in red) compared to controls (shown in blue). *p < .05. (For interpretation of the references to color in this figure legend, the reader is referred to the web version of this article.)

To investigate how HIV infection impacts sensory processing, we computed a 2 × 2 mixed-model ANOVA (stimulation-by-group) on response power. This analysis revealed a significant main effect of stimulation, such that there was a weakened response to stimulation 2 compared to stimulation 1 across all participants (p < .001). In other words, we observed significant somato-gating in the relative power envelope (Fig. 2). There was also a main effect of group, such that HIV-infected participants exhibited weakened somatosensory cortical responses to the stimulation compared to uninfected controls (*p* = .004; Fig. 2). There was also a trending stimulation-by-group interaction (*p* = .082).

Next, as described in the methods, we computed the gating ratio by dividing the peak power of the response to stimulation 2 by that of stimulation 1 in each participant. The resulting quotient, or gating ratio, reflects the individual's capacity to “gate” the second stimulus in an identical pair, with smaller values indicating stronger gating (i.e., better suppression of redundant stimuli). Independent-samples *t*-tests revealed no significant difference in gating between HIV-infected patients (*M* = 0.51) and uninfected controls (*M* = 0.53, *p* = .910).

**Fig. 3:**
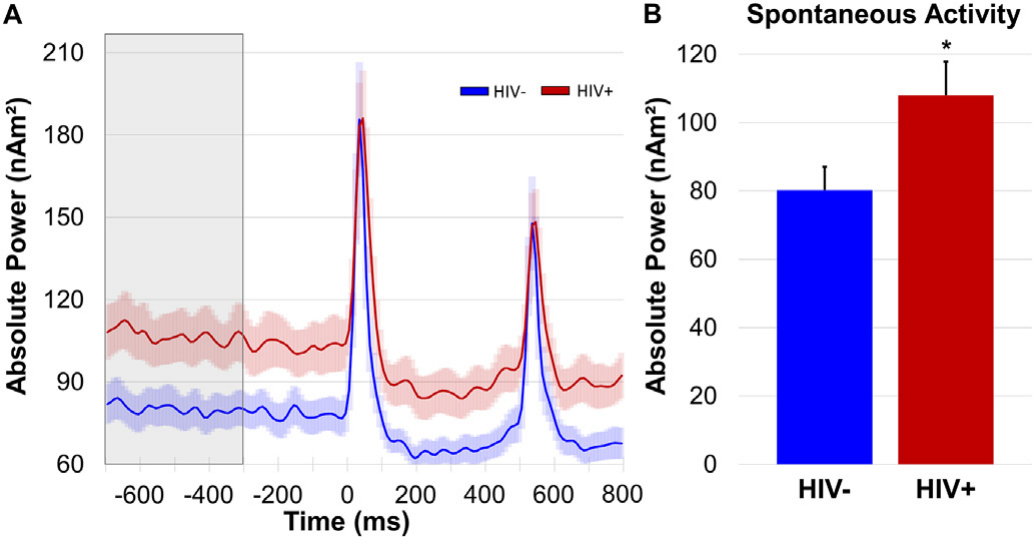
Spontaneous neural activity. (A): Absolute voxel time series extracted from the peak voxel and averaged across each group revealed elevated spontaneous neural activity immediately preceding stimulus onset in HIV-infected participants. (B): Independent samples t-tests revealed significantly elevated gamma activity during the baseline period (−700 to −300 ms) in patients with HIV (shown in red) relative to controls (shown in blue). *p < .05. (For interpretation of the references to color in this figure legend, the reader is referred to the web version of this article.)

Finally, to evaluate whether spontaneous neural activity immediately preceding stimulus onset differed as a function of group, we used the absolute power time series data (i.e., not baseline-corrected; Fig. 3) computed for the same voxels as were used in the relative analyses described above. We then calculated the mean power during the baseline period (−700 to −300 ms) and conducted an independent-samples t-test, which revealed that 20–75 Hz spontaneous gamma power was significantly stronger in HIV-infected participants relative to controls in the post central gyrus, *t*(66) = −2.14, *p* = .036.

### 3.4 Frequency-specific alterations in HIV-infection

**Fig. 4:**
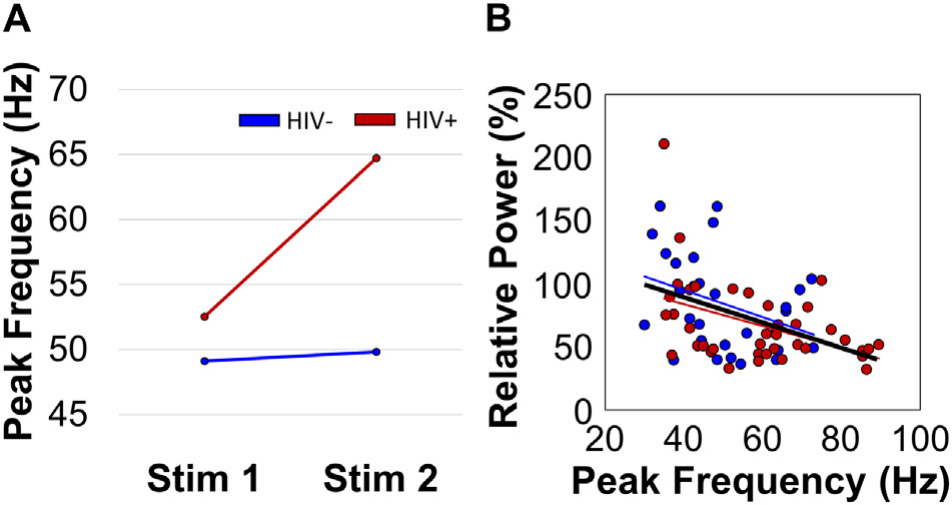
HIV-infection modulates peak gamma frequency in the somatosensory cortex. (A) A mixed-model ANOVA revealed a significant stimulation-by-group interaction, as well as main effects of group and stimulation. The interaction effect revealed that HIV-infected participants had a higher peak gamma frequency in response to the second stimulation relative to controls (*p* = .045), while the main effects indicated overall higher peak gamma in HIV-infected participants (group effect), and higher peak gamma in response to the second stimulation (stimulation main effect). (B) Pearson correlations revealed a significant association between peak gamma frequency and response power, such that increases in peak gamma frequency were linked with decreases in response power, irrespective of stimulation number (first or second) in patients with HIV (shown in red), uninfected controls (shown in blue), and across groups (shown in black). (For interpretation of the references to color in this figure legend, the reader is referred to the web version of this article.)

Given findings suggesting that the peak frequency is a key parameter of gamma responses and may be related to aging (Gaetz et al., 2011; Gaetz et al., 2012), a mixed model ANOVA (stimulation-by-group) was used to evaluate alterations in peak gamma frequency. This analysis revealed a main effect of stimulation, such that the peak gamma frequency was significantly higher for the second stimulation compared to the first across all participants (*p* = .025). There was also a main effect of group, such that HIV-infected participants had a significantly higher peak gamma frequency in the postcentral gyrus compared to uninfected controls (*p* = .010). Finally, there was a significant group-by-stimulation interaction, suggesting that compared to controls, HIV-infected participants had a higher peak frequency in response to the second stimulation compared to the first (*p* = .045; Fig. 4A). Lastly, to evaluate the overall relationship between peak gamma frequency and gamma power, we collapsed across groups and across stimulations (i.e., computed the mean peak frequency and power for each participant). Pearson correlations between these parameters revealed a significant association among peak gamma frequency and response power, such that as peak gamma frequency increased, response power decreased, *r*(67) = −0.44, *p* < .001, irrespective of HIV-infection or stimulation (Fig. 4B). For completeness, we also computed Pearson correlations between peak gamma frequency and response power for each group individually. This analysis revealed that the same significant association among peak gamma frequency and response power was present in HIV-infected participants, *r*(39) = −0.44, *p* = .004, as well as a trending association in the uninfected controls, *r*(27) = −0.35, *p* = .068 (Fig. 4B).

## 4 Discussion

The goal of the current study was to evaluate the impact of HIV-infection on pre-attentive inhibitory processing using a paired-pulse electrical stimulation paradigm that is known to elicit strong gamma oscillations in the postcentral gyrus. Through analysis of voxel time series data, we observed robust somatosensory responses in the gamma range (20–75 Hz) following right median nerve stimulation in uninfected controls, along with sharply reduced responses in HIV-infected participants. These oscillatory responses were used to compute a gating ratio and significant gating was observed across all participants, and surprisingly we observed no difference in gating as a function of group. In contrast, HIV-infected participants exhibited significantly elevated spontaneous neural activity prior to stimulation onset compared to controls, which may support theories of accelerated aging in HIV. Finally, given recent findings involving peak gamma frequency, we evaluated this metric and found altered peak gamma responses in HIV-infected adults. The implications of these novel findings are discussed below.

In the current study, we observed a disease-related reduction in gamma power in response to electrical stimulation. Previous studies using neuroimaging modalities such as fMRI and MEG have provided evidence for similar disease-related alterations in the primary visual, motor and somatosensory cortices (Ances et al., 2009; Ances et al., 2010; Wilson et al., 2013a; Wilson et al., 2015; Wilson et al., 2013b). Specifically, using fMRI, Ances and colleagues found reduced activity in the primary visual cortices during visual stimulation and at rest in HIV-infected adults (Ances et al., 2009; Ances et al., 2010). Similarly, Wilson et al. used tactile stimulation and a finger tapping experimental paradigm to investigate changes in oscillatory activity in HIV-infected participants. These studies revealed a reduction in theta and beta oscillations in the primary somatosensory and motor cortices, respectively, in HIV-infected participants compared to controls (Wilson et al., 2015; Wilson et al., 2013b). *En masse*, these findings and those of the current study support a reduction in primary sensory and motor responses across imaging modalities in HIV-infected adults. However, no neuroHIV study to date has evaluated such aberrations in the context of a sensory gating paradigm, which is thought to directly probe pre-attentive inhibitory processing.

As briefly described in the introduction, sensory gating is a process whereby the response to the second stimulus in a pair of redundant stimuli is attenuated. While this process has been extensively studied in the auditory domain with respect to schizophrenia and other psychiatric disorders (Cheng et al., 2016a; Light and Braff, 1999; Thoma et al., 2017), far fewer studies have examined such gating in the somatosensory cortices. Furthermore, this phenomenon had yet to be investigated with respect to the impact of HIV on the CNS. Nonetheless, contrary to our hypothesis, HIV-infected participants did not differ from uninfected controls in their ability to gate. This apparently normal capacity to gate redundant stimuli in HIV-infected adults may be related to alterations in their peak gamma frequency in response to the second stimulation. Briefly, we found that HIV-infected participants had a higher peak gamma frequency in response to stimulation, and that this effect was driven by an increase in the peak gamma frequency for the second, redundant stimulation in the pair. Further, regardless of group or stimulation, peak gamma frequency was significantly correlated with peak gamma power, such that as frequency increased, response power decreased across all individuals. This inverse relationship between peak gamma frequency and gamma response power has been widely reported in the literature (e.g., Wilson et al., 2018), and thus the sharp increase in peak frequency observed in response to the second stimulation in the HIV-infected group may have been critical to the decrease in power. In regard to mechanisms, considerable evidence suggests that such gamma oscillations are closely associated with inhibitory interneuronal networks (Bartos et al., 2007; Buzsáki and Wang, 2012; Fries, 2009; Fries et al., 2007; Salkoff et al., 2015), and multiple multimodal studies have linked local GABA concentration and/or local GABA-A receptor density to gamma response metrics (i.e., peak frequency and/or amplitude) in the motor and visual cortices of healthy adults (Gaetz et al., 2011; Kujala et al., 2015; Muthukumaraswamy et al., 2009). These multimodal studies have used MEG with either GABA magnetic resonance spectroscopy (MRS) or flumazenil-positron-emission tomography (PET) methods, and their findings have provided a mechanistic link between high-frequency gamma oscillations and local GABA concentration and/or receptor density. Thus, we propose that the increase in peak gamma frequency in response to the second stimulation in the HIV-infected group may have contributed to the attenuations in response power necessary for proper gating of somatosensory input, and that these effects were potentially GABA-mediated. However, we acknowledge that this interpretation is speculative and future work must directly investigate the directional flow of these relationships.

Another novel contribution of the current study is the investigation of spontaneous neural activity in HIV disease. To date, few studies have examined this concept, and almost all of those have probed the effects of healthy aging on spontaneous cortical activity. Briefly, Rossiter and associates were able to detect an age-related elevation of spontaneous beta activity during a pre-movement baseline period, with movement-related beta oscillations following a similar age-related trajectory (Rossiter et al., 2014). A more recent study using a finger-tapping task took this a step further and directly linked movement-related beta oscillations with baseline activity. Essentially, they found that both of these metrics influenced motor performance in healthy aging adults and that the amplitude of the beta motor response was contingent on the amplitude of the baseline period directly preceding it (Heinrichs-Graham and Wilson, 2016). Finally, a third study of healthy aging found that spontaneous gamma activity during the baseline directly modulated an age-related decline in the processing of somatosensory stimulations (Spooner et al., 2018). Taken together, these previous findings suggest an age-related increase in spontaneous neural activity, and that these elevations in baseline power are directly related to the event-related oscillations that serve primary motor and somatosensory function. Like previous work, we found alterations in spontaneous power prior to stimulation onset in the current study, but this increase was attributable to HIV-infection and not age (i.e., the two groups were closely matched on this variable). Similar findings were recently reported in the occipital cortices of HIV-infected patients (Wiesman et al., 2018). From these, we can conclude that increases in spontaneous neural activity are not only associated with healthy aging, but are also observed in conditions thought to be associated with premature or accelerated aging (e.g., HIV-infection). Future studies should further evaluate the link between accelerated aging and elevations in spontaneous activity in the context of neuroHIV. Given the normative aging data suggesting increases in spontaneous activity with age (see Heinrichs-Graham et al., 2018), metrics of spontaneous neural activity may be a useful avenue to map the overall trajectory of age-related decline in neuroHIV. Such analyses would also open up the possibility of identifying regional differences in the trajectory of age-related alterations.

## 5 Conclusions

In conclusion, the current study quantified disease-related alterations in pre-attentive inhibitory processing in the somatosensory cortex of HIV-infected adults. While participants with HIV did not differ in their gating of repetitive stimuli, other somatosensory indices were significantly altered. For example, our study showed that HIV-infected participants have a reduction in neural activity across the primary sensory modalities compared to uninfected controls, which is consistent with previous work (Ances et al., 2009; Ances et al., 2010; Wilson et al., 2015; Wilson et al., 2013b). We also found that spontaneous gamma activity was significantly elevated in HIV-infected adults compared to uninfected individuals, and that HIV-infected participants had a sharply elevated peak gamma frequency response to the stimulation, especially in regard to the second, redundant stimulation. This finding may reflect a potential compensatory mechanism in HIV-infected adults, which enables them to properly inhibit repetitive stimuli in the somatosensory domain, but further work is needed to clarify the mechanism and decipher the critical elements. Such oscillatory alterations in the somatosensory cortices have been detected in other disorders, including PTSD (Badura-Brack et al., 2015) and healthy aging (Cheng and Lin, 2013; Lenz et al., 2012; Spooner et al., 2018), both of which are major topics in the field of neuroHIV. Finally, with the growing evidence supporting accelerated aging in HIV-infected adults (Cole et al., 2017; Gross et al., 2016; Kuhn et al., 2017; Wade et al., 2015), it is imperative that future studies examine the lifespan trajectory of somatosensory function and gating in HIV-infected individuals.
